# Look for methods, not conclusions

**DOI:** 10.1038/s41419-019-2179-8

**Published:** 2019-12-05

**Authors:** Roberto Bassi, Enrico M. Bucci, Raffaele A. Calogero, Piero Carninci, Gennaro Ciliberto, Pellegrino Conte, Michele De Luca, Gilberto Corbellini, Antonio Giordano, Luigi Marchionni, Giacomina Massaro Giordano, Angelo Parini, Gianluca Sbardella

**Affiliations:** 1 Biotechnology Department, Università di Verona, Strada Le Grazie 15, 37134 Verona, Italy; 2 Accademia Nazionale dei Lincei, Roma, Italy; 3 Sbarro Health Research Organization, c/o Temple University, 1900 N 12th St -, 19122 Philadelphia, PA USA; 4 Department of Molecular Biotechnology and Health Sciences, University of Torino, Via Nizza 52, 10125 Torino, Italy; 5 RIKEN Center for Integrative Medical Sciences, Yokohama, Kanagawa Japan 230-0045; 6 IRCCS, Regina Elena National Cancer Institute, Rome, Italy; 7 Department of Agricultural, Food and Forest Sciences, University of Palermo, Viale delle Scienze, Building 4, 90128 Palermo, Italy; 8 Center for Regenerative Medicine “Stefano Ferrari,” Department of Life Sciences, University of Modena and Reggio Emilia, 41125 Modena, Italy; 9 Department of Molecular Medicine, “Sapienza” University of Rome, Rome, Italy; 10 Sidney Kimmel Comprehensive Cancer Center, Johns Hopkins University, 401 N Broadway, Baltimore, MD 21231 USA; 11 Department of Ophthalmology, Scheie Eye Institute, University of Pennsylvania, Philadelphia, PA USA; 12 INSERM, U1048, Toulouse, F-31432 France; 13 Université de Toulouse, UPS, Faculté des Sciences Pharmaceutiques, F-31062 Toulouse, France; 14 Department of Pharmacy, University of Salerno, Fisciano, I-84084 Italy

**Keywords:** Molecular biology, Diseases

Dear Editor,

Honest errors, sloppy practices or true frauds: there can be several causes of inconsistences and problematic data in a scientific paper. However, whatever the actual outcome of an investigation into a case of alleged data manipulation, there are certain firm principles that always hold true, and certain errors that must be avoided.

In the most recent, high-profile case of allegedly manipulated images in scientific papers, we learnt from the principal investigator that irrespective of the investigation outcomes and of any actual manipulations:

“*I remain confident about the validity and strength of the scientific conclusions made in those publications*.”^1^

Nearly all scientists accused of data manipulation come up with similar words; more worryingly, such a view recently seems also to be shared by those appointed to judge cases of potential misconduct.

While it might be true that the scientific conclusions of some research papers under investigation will eventually appear supported by overwhelming scientific evidence, we take this occasion to recall a few concepts that seem to be at stake every time the focus shifts from investigating the evidence for data manipulation to assessing the theories those data were intended to support.

Pretending that erroneous or fraudulent data manipulation is more acceptable if, retrospectively, it does not compromise the validity of the main conclusions of a scientific work, involves accepting that sloppy or deceptive practices are forgivable or less serious, if the hypothesis one intends to support eventually proves right. This is equivalent to accepting a scientist’s educated guess, instead of relying on actual experimental data.

Such an attitude is radically opposed to the scientific method and the ethics of scientific knowledge according to Jacques Monod. The ethical commitment of a scientist is to produce and communicate objective facts. Double standards cannot be accepted, neither by those who allegedly fabricated, falsified or duplicated experimental data, nor by those assessing the seriousness of a suspected manipulative behaviour, for the following reasons:

1. It undermines the trust in the data produced by researchers, and so the trust in science itself.
2. It brings back science to the speculative approach of the premodern world, well before the experimental method transformed the investigation of the natural world; back then theories were formulated mainly based on a purely deductive approach, from arbitrarily selected first principles.
3. It shifts the examination of a potential misconduct case from the assessment of the actual behaviour and responsibility of the people involved, to the validity of the theory they happened to disclose to the scientific community.
4. It confers an unfair advantage onto those who, in the current, prevalent publish-or-perish competition, have few if any actual experimental evidence, but fill the gaps with improperly duplicated or manipulated data.

The duplication or the alteration of data in a published paper might be due to honest error, and a research group, including the principal investigator, might on occasion have been fooled by a single, dishonest or sloppy researcher. But once a duplication, a falsification or a fabrication is discovered in a published paper, one cannot appeal to the validity of the scientific conclusions of a paper to avoid or delay one of the main duties of the researcher: which is to correct the published record. This must follow a thorough and honest disclosure of all the potential problems and biases that caused the leakage of less-than-clean data into a scientific publication, in order to reassure peers, institutions and the public that every reasonable system to avoid further problems is in place.

The relentless defence of duplicated, fabricated or falsified data is, per se, a form of serious misconduct, which transforms even an honest error into pertinacious misbehaviour, and honesty must be prized at least as intelligence and knowledge, to ensure that science corrects itself.
